# Non-Canonical Aspects of Antibiotics and Antibiotic Resistance

**DOI:** 10.3390/antibiotics13060565

**Published:** 2024-06-17

**Authors:** Carlos F. Amábile-Cuevas, Sofia Lund-Zaina

**Affiliations:** 1 Fundación Lusara, Mexico City 08810, Mexico; 2 Department of Public Health, University of Southern Denmark, Campusvej 55, 5230 Odense, Denmark

**Keywords:** antibiotic resistance, horizontal gene transfer, antibiotic usage, socioeconomic factors, disinfectants, mechanism of action, mechanism of resistance

## Abstract

The understanding of antibiotic resistance, one of the major health threats of our time, is mostly based on dated and incomplete notions, especially in clinical contexts. The “canonical” mechanisms of action and pharmacodynamics of antibiotics, as well as the methods used to assess their activity upon bacteria, have not changed in decades; the same applies to the definition, acquisition, selective pressures, and drivers of resistance. As a consequence, the strategies to improve antibiotic usage and overcome resistance have ultimately failed. This review gathers most of the “non-canonical” notions on antibiotics and resistance: from the alternative mechanisms of action of antibiotics and the limitations of susceptibility testing to the wide variety of selective pressures, lateral gene transfer mechanisms, ubiquity, and societal factors maintaining resistance. Only by having a “big picture” view of the problem can adequate strategies to harness resistance be devised. These strategies must be global, addressing the many aspects that drive the increasing prevalence of resistant bacteria aside from the clinical use of antibiotics.

## 1. Introduction

There is a canonical way of looking at antibiotics: they are “magic bullets”—a colloquialism used by Ehrlich to describe selective toxicity—which are mainly used as therapeutic agents against bacterial infections. They act, and do so selectively, by affecting structures, enzymes, or biochemical pathways that are found only (or mostly) in prokaryotic cells. In turn, these effects result in the inhibition or death of bacteria exposed to minute concentrations of the drugs. Most antibiotics in clinical use are obtained from other microbes, typically soil bacteria, that release these compounds as “chemical weapons” to ward off competition. Antibiotics exert their inhibitory effect starting at a given concentration (the minimal inhibitory concentration, MIC), which should be surpassed and maintained within the patient to cure the infection. The extent and effects of exposure to sub-inhibitory concentrations are of minimal importance in terms of clinical efficacy.

Bacterial resistance to antibiotics can also be looked at from a canonical point of view. A bacterial strain is deemed “resistant” to a given antibiotic if it can multiply in the presence of clinically attainable concentrations of the drug. Resistance can be assessed by simple laboratory assays. Their results, MICs or inhibitory haloes (when using disks impregnated with antibiotics) are checked against “breakpoint” tables that define “susceptibility” and “resistance”: if an MIC is above (or the inhibitory halo diameter is below) the corresponding breakpoint, the isolate is considered resistant, implying that such treatment will fail. Inherently, resistance is only considered a cause for concern when it is found in pathogenic bacteria—and so it is almost only defined and searched for among such organisms. Genes responsible for a resistance phenotype are named “resistance genes” and included in databases used for metagenomic assessments. Resistance was thought to emerge only via chromosomal mutations, but it was soon discovered that resistance genes could be transferred between bacterial cells. This happens by the uptake of free DNA, carried by bacteriophages or, mostly, by plasmid-mediated conjugation (horizontal gene transfer, HGT); either way, formerly susceptible bacteria suddenly become resistant. Under the selective pressure posed by the clinical use of antibiotics, resistant varieties thrive and replace susceptible populations. Curtailing antibiotic usage can therefore reduce the selective pressure and, eventually, the prevalence of resistant isolates. Antibiotic stewardship is therefore crucial to cope with bacterial resistance, while research and development of new antibiotics is “jump-(re)started” through incentives for pharmaceutical companies. As these basic concepts are common for both developed and developing countries, strategies to face resistance should be the same globally. The notions above can be found in many textbooks on the matter (e.g., Ref. [1]).

Most of these concepts are only partially true (and some are plain myths [2]), but they are still at the core of mainstream knowledge on antibiotics and resistance, especially in clinical settings. (Amongst lay people, this knowledge is much worse, with wrong notions accepted by many, such as antibiotics being useful against viruses, or resistance being caused by not finishing antibiotic treatments [3].) As resistance has mainly clinical consequences, it is particularly concerning that many aspects of antibiotics and resistance are still missing from the “common knowledge” among clinicians. Moreover, as decisions on facing resistance fall mostly on clinicians, it is crucial that they have realistic views of the origin and spread of antibiotic resistance. The purpose of this review is to bring these non-canonical perspectives to the attention of clinicians and others familiar only with outdated views of antibiotics and resistance.

## 2. Antibiotics

The term “antibiotic” has many definitions, from the very lax “a medicine that inhibits the growth of or destroys microorganisms” (*Oxford Dictionary of English* online) to the notion of “small molecules” that are “natural chemotherapeutic agents” [4]. Here, we refer to “antibiotics” as natural or synthetic molecules that, due to their selective toxicity, can be used to treat bacterial infections affecting animals and even plants. The actual role of natural antibiotics in microbial physiology and ecology is still debatable. Whether they are the “chemical weapons” of microbial wars or the remnants of prebiotic chemistry [5], now used for interbacterial communication or bacteria–eukaryote symbiosis, it is important to realize that bacteria have been producing and interacting with antibiotics for billions of years, albeit in minute amounts [6,7]. Some synthetic antibiotics even mimic the effects of ancient natural molecules: quinolones have similar effects to chloroquine [8] and novobiocin [9], and oxazolidinone antibiotics (e.g., linezolid) inhibit bacterial protein synthesis by attaching to the same ribosomal site as the natural antibiotics chloramphenicol, lincomycin, and pleuromutilin [10]. It is therefore not surprising to find resistance determinants, even against synthetic drugs, among ancient bacterial genes, but we must acknowledge that the current use and abuse of antibiotics is not the origin of most clinically relevant resistance traits.

### Antibiotics’ Mechanisms of Action

**Figure 1:**
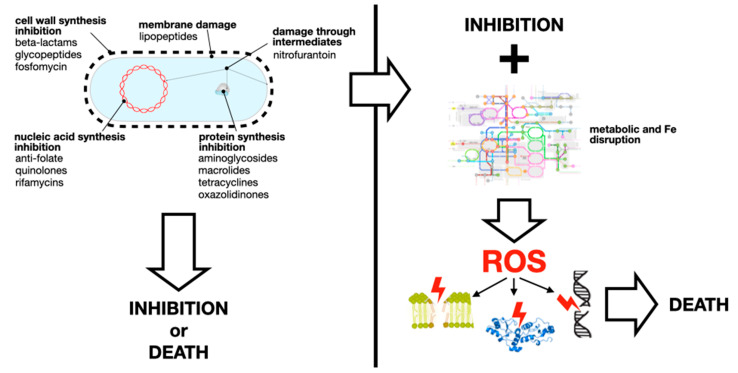
**Canonical and non-canonical mechanisms of action of antibiotics.** (**Left**): The classical mechanisms of action of antimicrobial drugs, such as protein synthesis inhibition or membrane damage, cause growth inhibition and/or cell death by themselves. (**Right**): In bactericidal antibiotics (e.g., beta-lactams, aminoglycosides, and fluoroquinolones, although the line between -cidal and -static is blurred), these mechanisms cause growth inhibition and disruption of metabolism and/or iron storage; the latter causes an increase in intracellular reactive oxygen species (ROS), which, in turn, damage DNA, proteins, and membranes. Such damage could be responsible for cell death.

Figure 1 (left) shows a summary of the known (or “canonical”) mechanisms of action of antibiotics. For instance, the inhibition of cell wall synthesis supposedly causes bacterial cells to be partially devoid of wall during cell division, which results in water uptake, bursting the bacterial cell in a hypotonic environment. Although there is unsurmountable evidence of antibiotics acting as described in Figure 1 (left), the actual cause of bacterial death by “bactericidal” antibiotics has been a matter of recent debate. Free radicals, generated by the disruption of bacterial cell physiology by canonical mechanisms, have been proposed as responsible for the bactericidal effect many antibiotics have. Dwyer et al. [11] cite extensive evidence of reactive oxygen species (ROS) being involved in the killing of bacteria under antibiotic treatment: direct or indirect detection of ROS, or of oxidative byproducts that could, in turn, have toxic effects; protection by, and/or induction of antioxidative defense systems, etc. Newer, indirect evidence includes the antibiotic-induced switching to anaerobic metabolism, so that ROS production is reduced [12], and the restoration of carbapenem susceptibility of carbapenemase-producing Escherichia coli by adding oxidized glutathione, which impairs the antioxidant status of the bacterial cell [13]. 

As with other biological phenomena related to ROS, controversy has arisen due to the elusive nature of free radicals. Liu and Imlay [14], for instance, found that the bactericidal effect of antibiotics persisted even in the absence of oxygen, and that mutants lacking DNA repair mechanisms were not more susceptible to the drugs. Also, Hassett and Imlay [15] discussed the killing of bacteria even under low-oxygen conditions (e.g., biofilms), suggesting that ROS “might contribute to toxicity, but [are not] essential” to the bactericidal effect. Genes known to confer resistance to oxidative stress could indeed confer resistance to antibiotics; however, the reasons for which such defense mechanisms can also protect bacteria against antibiotics might be unrelated to oxidative stress. For instance, the *soxRS* regulon of *E. coli*, which is induced by superoxide and nitric oxide, includes antioxidant defense and repair mechanisms, as well as mechanisms that can reduce the intracellular concentration of many non-oxidative xenobiotics, simply by reducing outer membrane permeability and increasing unspecific efflux [16]. Likewise, *Klebsiella pneumoniae* strains lacking SoxS have diminished efflux, allowing for the build up of intracellular antibiotics concentration, thereby reducing the MIC [17]. If some antibiotics do, indeed, induce ROS production inside bacterial cells, bacteria resistant to such antibiotics should also have increased resistance to oxidative stress. In a survey of *E. coli* isolates, those with higher resistance to hydrogen peroxide tended to be resistant to more antibiotics, particularly the bacteriostatics sulfadiazine and tetracycline; additionally, superoxide resistance was associated with beta-lactam resistance. But the relationships between antibiotic and pro-oxidant resistance were too weak to support the hypothesis of ROS involvement [18]. Ultimately, the distinction between bacteriostatic and bactericidal antibiotics is merely academic, as it is only based on in vitro experiments, can vary between bacterial species for a single drug, and has no obvious clinical relevance [19,20]. Furthermore, drugs in the same class could have different dependence on ROS for their bactericidal effect. For instance, norfloxacin, a second-generation quinolone, depends on ROS for bacterial killing at low concentrations, while ciprofloxacin, a third-generation one, is less dependent on ROS [21]. This obscures the involvement of ROS in antibiotic activity.

Nevertheless, ROS being either crucial or secondary effectors of antibiotic activity could have important clinical repercussions. Antioxidants could hamper the effect of antibiotics, perhaps even causing bacterial tolerance or persistence (see below). Actual evidence of this happening is scarce and in vitro only: bipyridyl and thiourea diminished the bactericidal effect of norfloxacin [22], and antioxidant polyamines protected against beta-lactams, aminoglycosides, and fluoroquinolones [23]. It is, however, conceivable that increasing the levels of antioxidants (e.g., vitamin E, capable of reducing the lethality of paraquat, a superoxide-generating agent [24]) could reduce the clinical efficacy of antibiotic treatments. Also, agents or conditions that induce bacterial responses to oxidative stress could reduce the therapeutic efficacy of antibiotics, from salicylate, a metabolite of aspirin, which induces Mn–superoxide dismutase through *E. coli*’s MarA regulon [25], to the “oxidative burst” of macrophages that induces *E. coli*’s *soxRS* regulon [26]. Sublethal levels of ROS resulting from mutations in the defense gene *soxRS* resulted in more rapid acquisition of resistance to the bactericidal antibiotics amoxicillin, enrofloxacin, and kanamycin (but not bacteriostatic tetracycline) [27]. These observations reveal metabolic–antibiotic synergies and antagonisms that require further examination for novel therapeutic targets and approaches.

## 3. What Is Resistance?

Most papers and books on antibiotic resistance (AR) start by presenting the clinical impact of resistant bacteria, assuming that there is a universal definition of “resistance”. Clinically, a bacterial strain is deemed “resistant” to a certain antibiotic if the antibiotic’s MIC is high enough to cause the drug to fail when used to treat an infection caused by the strain. The MIC breakpoints established by several organizations (e.g., Clinical and Laboratory Standards Institute, CLSI, in the US; Eucast in the EU) are different for each antibiotic, bacterial group, and even affected body site. Sometimes, these organizations disagree in their breakpoints, so a given strain can be “susceptible” in Europe, and “resistant” or “intermediate” in the US or vice versa; for instance, a *Pseudomonas aeruginosa* strain with a colistin MIC of 4 µg/mL is susceptible in Europe and resistant in the US. Nonetheless, the breakpoints are meant to be used for clinical purposes. From a simple susceptible/resistant dichotomy (and the even more vague “intermediate” category), there now are also categories like “susceptible-dose dependent”, “nonsusceptible”, “epidemiological cut-off values” (ECOFFs), and “area of technical uncertainty”, adding confusion to issues like resistance prevalence. Furthermore, there are no breakpoints for non-clinically relevant bacteria, nor for antibiotics that are not used clinically against a given pathogen; resistance in these two cases cannot be easily defined. This has not stopped researchers from using clinical breakpoints to characterize resistance among environmental bacteria, or even worse, from using entirely arbitrary antibiotic concentrations to select for resistance in the laboratory. For instance, many papers report isolating resistant enterobacteria by plating samples on media containing 50–100 µg/mL of ampicillin (for which the resistance breakpoint is ≥32 µg/mL), or 50 µg/mL of streptomycin (for which there are no MIC breakpoints, as streptomycin is not used clinically against enterobacteria). The relevance of the data obtained in this way is not always clear.

A separate paragraph must be devoted to the MIC. This pharmacodynamic parameter, on which the definition of resistance relies, has many limitations, starting with its in vitro assessment in rich culture media, which is very different from in vivo conditions (see also Box 1). The effects of antibiotics on bacteria in different growth stages, cell densities, and nutritional environments cannot be predicted by the MIC obtained in Mueller–Hinton broth with a standardized inoculum, nor can they reflect the actions of different drugs, despite having similar MICs. “The time has probably arrived to produce a more complex way of predicting the activity of an antibiotic on a bacterial population” [28].

Box 1 **Susceptibility testing (the antibiogram).**

  The value of knowing if a microorganism causing an infection is susceptible to a given drug is obvious: if it is, the treatment will likely be successful, if it is not, the treatment will likely fail. The reliability of this information is also obviously crucial: if a microorganism is wrongly deemed “susceptible”, the wrong antibiotic will be used, resulting in failure; if it is wrongly deemed “resistant”, the right antibiotic will not be used, resulting in the likely escalation to unnecessarily newer, more “potent”—and often more expensive—drugs. This is the case both for individual tests to select the treatment for a single patient, and for empirical treatment based on epidemiological data, in turn based on many individual tests. Yet, as crucial as this information is to guide the millions of daily antibiotic prescriptions worldwide, we are still using the serial dilution method that Fleming introduced, along with penicillin, in 1929, or the diffusion methods (discs or strips) introduced in 1944 and standardized by Bauer et al. in 1966—the “Bauer–Kirby” method [29]. One result of this is the informal “90–60 rule”: if an organism is susceptible, there is a 90% probability of success, but if it is resistant, treatment will still succeed in 60% of the cases [30]. There are many shortcomings in typical susceptibility assays, such as their deliberately monoclonal nature, and the reductionist definition of resistance that guides them [31]. In addition, quality problems have been reported with in vitro diagnostics supplies (e.g., Ref. [32]) and divergent results yielded by different commercial assays (e.g., Ref. [33]). Entirely different approaches, such as searching for mobility genes strongly linked to AR (e.g., the integron integrases *intI1* and *intI2*), have shown better predictive capabilities: integrase detection has a predictive value of 92% for resistance to first-line antibiotics and 96% for resistance to third-generation cephalosporins [34]. With antibiotic resistance being a major public health threat, and with so many advances in biotechnology, it is inexcusable that crucial information, i.e., resistance prevalence, is obtained through dated, error-prone methods. It is also important for clinicians to understand such limitations and take the antibiogram results as only partially reliable.

Most attention is given to “acquired” resistance, which is gained through mutations or HGT, distinctly different from “intrinsic” resistance, which defines the original spectrum of each antibiotic (and that will not be further discussed here). Phenotypically, acquired resistance can be seen as a “jump” from low MICs, well below the resistance breakpoint, to values well above such breakpoint. This kind of dramatic change, which is common among horizontally transferred resistance traits, facilitates the discrimination between susceptibility and resistance. However, there are many examples of resistance phenotypes that are not that clear; most of them are caused by mutations in housekeeping genes. For instance, single mutations in the *gyr* or *par* genes, encoding different topoisomerases that are inhibited by fluoroquinolone drugs, confer only slight increases in MIC, but still below the resistance breakpoint [35]. (A horizontally transferred group of genes, *qnr*, also confers only a minor MIC increase [36].) Hence, strains carrying such single mutations (or *qnr* genes) are phenotypically classified as “susceptible” based on routine lab assays; paradoxically, *qnr* genes are classified as “resistance genes”. *gyr/par* mutations, *qnr* genes, and/or other genetic or physiological changes that reduce the effect of the drugs can accumulate in a single cell, bringing it to a full resistance phenotype [37,38]. This “grey” scenario is common for mutations in penicillin-binding proteins (PBPs), which reduce the effect of beta-lactams, as well as for changes in the intracellular accumulation of non-specific antibiotics caused by modifications in porins or efflux systems [39]. Establishing precise breakpoints is difficult under these conditions, and the clinical relevance of those established is not clear, muddling the definition of resistance.

A gradual increase in MIC within the range designated as sensitive has been named “MIC creep”; it has been reported mainly in staphylococci and enterococci, and particularly towards vancomycin (although at least one paper has described it in *Clostridoides difficile* [40] and another in *Neisseria gonorrhoeae* [41]). The increase in MIC demands higher doses of the antibiotic. While this nuance could be purely related to the type of assay used to assess susceptibility [42], and some authors even fail to find it [43], the designation could also fit phenomena like the increased fluoroquinolone MIC discussed in the paragraph above. Again, defining resistance is difficult in the light of so much variability.

If defining “resistance” is challenging, the definition of a “resistance gene” is much more so. It may sound obvious that a gene responsible for phenotypic resistance should be deemed a “resistance gene”. This rationale has been used to construct databases of such genes (e.g., Comprehensive Antibiotic Resistance Database [CARD], ResFinder, or AMRFinder), which aim to guide researchers from metagenomic analyses to diagnostic methods [44]. However, such strategies tend to group as equally relevant, the gene encoding an antibiotic-inactivating enzyme that can be horizontally mobilized among very different organisms, conferring almost always phenotypic resistance; and the housekeeping, chromosomal gene that, when overexpressed due to transient stress or to mutations in regulatory mecanisms, confer mild resistance that is almost never transferable, and cannot confer resistance in a genetic background different of its own. For instance, among the most frequent “resistance genes” in the CARD there are non-specific efflux pumps, porins with reduced permeability, and resistance to clinically irrelevant elfamycin [45]. The results of analysis using these databases usually disagree with each other, along with the sequencing methods used to provide substrate to such studies [46]. Conclusions based on metagenomic analyses using these databases would not accurately inform of the real threat of resistance in conditions such as wastewater management [47]. In the clinical environment, while potentially useful for surveillance purposes, whole-genome sequencing is still unusable for diagnostics, as a high number of purported “resistance genes” are detected in otherwise phenotypically susceptible isolates [48]. Furthermore, a metagenomic analysis found that more than 80% of purported AR genes are not carried by plasmids or integrons, “highlighting the need to differentiate genes of high clinical relevance” [49]. Overall, metagenomic surveys that do not assess the actual risk of purported “resistance genes” causing antibiotic failure in the clinic, or migrating from harmless and/or environmental bacteria into human pathogens [50], are of little help in understanding the real magnitude of the resistance threat.

## 4. Non-Canonical Resistance: Hetero-Resistance, Tolerance, Persistence, and Even Dependence

While canonical resistance always involves an irreversible increase in antibiotic MICs, several other phenotypes may result in the therapeutic failure of an antibiotic without affecting the MIC or doing so only transiently (some authors refer to the former as “genotypic resistance” and the latter as “phenotypic resistance”, with possible clinical strategies for each one [51]). The current burden imposed by these other phenotypes, in terms of morbidity and mortality of bacterial infections, is nearly impossible to calculate, as there are no data on their prevalence. Therefore, they are neglected in most epidemiological considerations, as well as in the official definitions of “resistance”, such as the simplistic one by the WHO, as something that occurs when bacteria “change over time and no longer respond to medicines making infections harder to treat” (https://www.who.int/news-room/fact-sheets/detail/antimicrobial-resistance, accessed on 9 May 2024). This neglect extends to the development of easy assays that could enable clinical labs to detect these phenotypes, and of strategies to circumvent them during antibiotic treatment.

**Figure 2:**
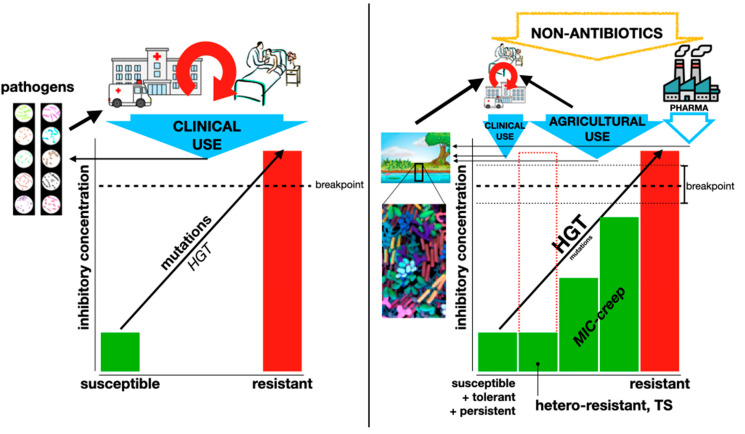
**Canonical and non-canonical concepts of resistance.** (**Left**): In the canonical view, bacteria are classified as susceptible or resistant, depending on the minimum inhibitory concentration (MIC) of a given antibiotic; if the MIC surpasses an established breakpoint, the isolate is deemed “resistant”. This classification has clinical purposes: if the antibiotic is used against an infection caused by a susceptible organism, there will be clinical success; if used against an infection caused by a resistant one, treatment will fail. The “jump” from susceptible to resistant is often caused by mutations, with horizontal gene transfer (HGT) playing an important yet secondary role. The main selective pressure for resistance is the clinical use of antibiotics, either in hospitals or outpatients (with resistant organisms cycling between these two scenarios). The pressure acts solely upon pathogens. (**Right**): The assessment of inhibitory concentrations alone classifies a number of phenotypes that can cause treatment failure as “susceptible”. For example, low MICs could include tolerant or persistent organisms; low MICs with little growth, often mistaken as contamination, could indicate hetero-resistance or “transiently silent” (TS) resistance; and an increase in MICs but remaining below the breakpoint is sometimes referred to as “MIC creep”. Breakpoints are increasingly blurred, making it difficult to classify an isolate as resistant or susceptible. The “jump” towards resistance is mainly driven by HGT rather than chromosomal mutations. While the clinical use of antibiotics is clearly relevant, most of the world’s production of antibiotics are used agriculturally. Both clinical- and agricultural-used antibiotics end up in the environment, mostly released in wastewater and manure, along with antibiotics released by pharmaceutical factories in some countries. (Some resistant organisms go directly back into foodstuff, causing clinical problems.) Additionally, a number of non-antibiotic selective pressures (e.g., disinfectants, non-antibiotic drugs) are also at play. Their effects are exerted not only on clinically relevant bacteria but also on the whole planetary microbiota, where resistance genes have been for millions of years. Through HGT, such genes can now travel back to pathogenic bacteria, making them resistant to old and new drugs.

Perhaps the best review on these phenotypes is the one by Schrader et al. [52]. From its very title, it proposes that canonical resistance (referred to as “genetic MIC-shifted resistance”), hetero-resistance, tolerance, and persistence should be seen as “a continuous spectrum of manifestations” rather than separate phenomena (Figure 2). The many conditions that can elicit any of these phenotypes (see below) further complicate the full understanding of bacterial resistance. However, it is necessary to incorporate all this knowledge into the clinical management of resistance, either as a problem affecting a single patient or as a public health issue. 

Additionally, a number of non-canonical resistance mechanisms have recently been grouped under the proposed denomination of “transiently silent acquired” resistance. This refers to genes acquired by mutation or HGT that are not phenotypically expressed until something changes within the bacterial cell. This can occur during the treatment of an infection deemed susceptible to a given antibiotic by routine testing and could result in treatment failure. It can easily be confused with hetero-resistance and adaptive resistance, discussed above, but it has distinctive features. However, they do have in common an added difficulty for assessment in routine susceptibility assays [53]. Another recently described phenotype called “perseverance”, very similar to hetero-resistance but detectable only in cell-to-cell comparison assays, affects the in vitro activity of at least nitrofurantoin and rifampicin [54].

Yet another rather extreme phenotype, dependence, adds to the confusion of non-canonical resistance. This phenomenon involves an antibiotic significantly fostering the growth of the affected strain, even to the point that the strain cannot grow without the drug. Dependence was found early after the discovery of antibiotics themselves. However, it was not until an *Enterococcus faecalis* strain, isolated from urine but failing to grow on subculture, and found to require vancomycin [55] that dependdence gained some notoriety. In addition to vancomycin, dependence of staphylococci on linezolid and of *Acinetobacter* spp. on colistin has recently been reported. The clinical impact of this phenotype has not been adequately addressed; withdrawing the antibiotic involved could help in curing the infection, unless spontaneous reversion to simple resistance occurs [56]. These kinds of organisms could be difficult to isolate in the laboratory, as most primary-isolation media do not contain antibiotics, and only little and slow growth would be achieved.

## 5. Non-Canonical Mechanisms of AR

**Figure 3:**
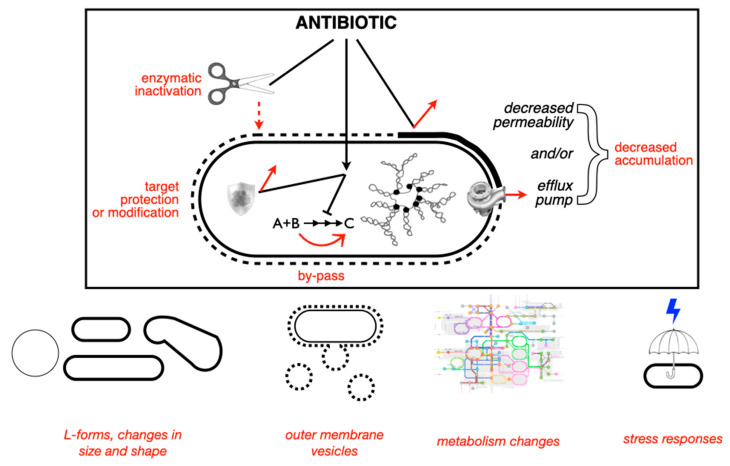
**Canonical and non-canonical mechanisms of AR.** (**Top**): The most recognized mechanisms of AR: enzymatic inactivation (represented by a pair of scissors), affecting beta-lactams, aminoglycosides, amphenicols, macrolides, tetracyclines, and fluoroquinolones; target protection or modification (represented as a shield protecting the ribosome, a usual target, but not the only one), affecting macrolides, tetracyclines, beta-lactams, quinolones, glycopeptides, lipopeptides, oxazolidinones; bypass, mostly referred to folate synthesis inhibitors (sulfonamides, trimethoprim), where an inhibited enzyme within the synthesis path is replaced by a resistant version; and decreased accumulation, resulting from decreased permeability and/or efflux pumps, affecting tetracyclines, macrolides, anfenicols, and quinolones (modified from Ref. [59]). (**Bottom**): Some non-canonical mechanisms of resistance: change in cell shape or form; increased presence of L-forms; generation of outer membrane vesicles; metabolism changes; and different stress responses.

There are plenty of reviews on the canonical mechanisms of antibiotic resistance, from one of the earliest by TJ Foster [57] to one of the latest by Darby et al. [58]. A summary of such mechanisms is shown in Figure 3. This section provides some examples of non-canonical mechanisms of canonical resistance, i.e., bacterial changes other than the typical enzyme inactivation or target modification that result in increased antibiotic MICs. Also, there are non-canonical mechanisms of non-canonical resistance. They are important, among other things, because they are likely to escape detection by molecular or bioinformatic methods focused on well-known resistance mechanisms or genes. Also, becasuse these few examples illustrate that bacteria can escape the effect of noxious chemicals by a much wider variety of mechanisms than the ones we usually think of.

Resistance acquired by antibiotic-specific efflux pumps, such as those exporting macrolides (*mef* genes) or tetracycline (early *tet* genes), are canonical mechanisms known for many years. Non-specific efflux of xenobiotics, such as the one mediated by the AcrAB-TolC family in enterobacteria, is also known to cause low-level multi-resistance when overexpressed, due to stress induction or mutations [39]. These efflux systems, however, are typically confined to vertical inheritance, as they are housekeeping traits. Importantly, “efflux pumps are ancient, highly conserved determinants, which have been selected long before the recent use of antibiotics [suggesting that their role] as relevant antibiotic resistance determinants […] is a recent event, likely secondary to other functional roles” [60]. However, recent reports indicate that gene clusters encoding complex efflux systems can be found in conjugative plasmids. TMexCD-ToprJ, for instance, mediates tigecycline resistance; it originates from *Pseudomonas* spp. (which are intrinsically resistant to tetracyclines), but it has been found in at least one enteric bacteria (*Raoultella ornithinolytica*) in a plasmid also carrying a carbapenemase gene [61]. Interestingly, due to the high energy consumption of these efflux pumps, mutations that overexpress them are amongst the only AR genes that tend to fade away in the absence of antibiotics [62]. Along with efflux, diminished permeability, also results in a reduced cytoplasmic antibiotics concentration. Changes in factors that affect the influx (e.g., porins, outer membrane composition) and/or efflux of antibiotics can impact the development of persistence and hetero-resistance [63]. Reductions in intracellular antibiotic concentrations are on the borderline between canonical and non-canonical resistance mechanisms. Table 1 lists some examples of non-canonical resistance mechanisms.

## 6. Non-Canonical Selective Pressures and Consequences

**Figure 4:**
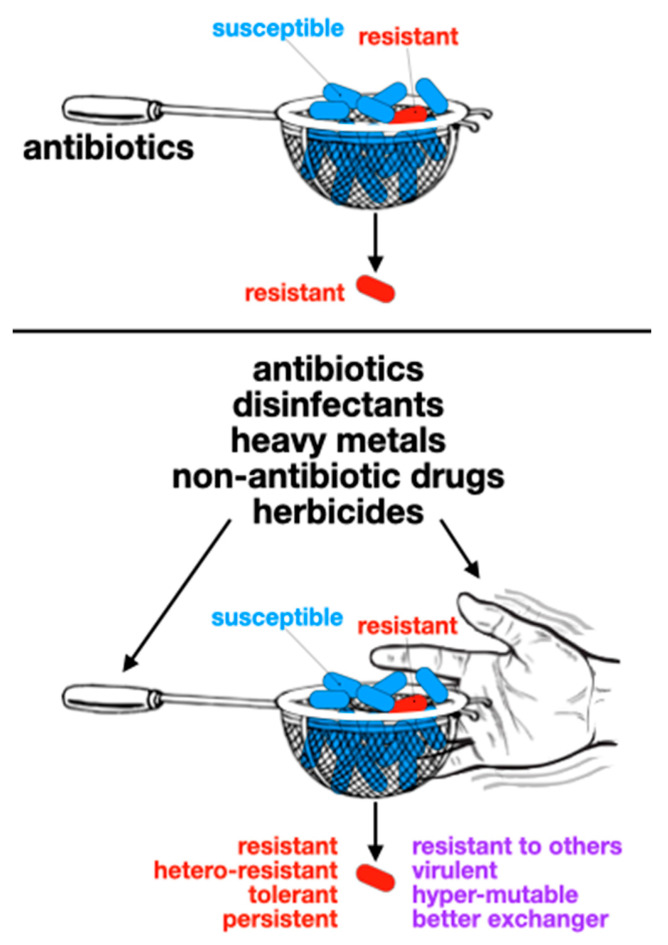
**“Sieving” resistant organisms from bacterial populations.** (**Top**): The canonical view of antibiotics acting as the only selective pressure (represented as a sieve) that allows for the “passage” of resistant bacteria, with resistance being the only trait selected for. (**Bottom**): In fact, many other agents can act as selective pressures (the sieve), facilitators (the hand tapping the sieve) for the acquisition of traits useful to survive the pressure, or both (some examples in Table 2). The selected organism may be canonically resistant or have phenotypes that enable surviving the selective pressure (i.e., hetero-resistant, tolerant, persistent, etc.). In addition to “resistance” towards antibiotics, other traits might slip through the selective sieve along with antibiotic resistance.

The obvious selective and maintenance pressure for AR determinants is antibiotic presence. With increasing (although disparate) use of antibiotics worldwide (from 9.8 defined daily doses (DDD) per 1000 inhabitants in year 2000 to 14.3 in 2018, ranging from 5.0 in the Philippines to 45.9 in Greece [75]), it is easy to conclude that rising AR is merely the consequence of antibiotic usage. The massive production and release of antibiotics during the early “antibiotic era” was likely related to the emergence and spread of resistance traits among pathogenic bacteria that were rather rare before. This was initially concluded from a comparison between clinical isolates from the “pre-antibiotic era” (the Murray Collection) and contemporary isolates [76]. Then, a number of reports correlated antibiotic usage and AR at the country level, indicating, again, that antibiotic usage was and is the main selective pressure for resistance (e.g., Ref. [77]). However, further studies on this topic found that the correlation was rather weak, and that other factors were contributing more directly. Non-biological issues affecting resistance prevalence will be discussed in another section of this article; here, we will focus on non-antibiotic agents or conditions that select for AR, and the consequences of this selection (Figure 4). Most studies focus on non-antibiotic antimicrobial agents, such as disinfectants (e.g., quaternary ammonium compounds (QACs), chlorhexidine, triclosan) and heavy metals (e.g., mercury, copper, silver [78]). But many other agents without antimicrobial activity are also capable of affecting bacterial responses to antibiotics; the list of “emerging contaminants” contributing to the AR problem is extensive [79]. Importantly, bacteria are routinely exposed to many agents that are toxic to them: antiseptics, sterilants, disinfectants of different potencies and spectra, and preservatives [80]. This exposure increased dramatically during the COVID-19 pandemic, with impacts on AR yet to be known [81]. The mechanisms of action of these biocides, as well as most mechanisms of resistance, are entirely different from those of antibiotics [82].

Co-selection is the first obvious culprit. The linkage of antibiotic resistance genes with other traits would make the pressures selecting for such other traits capable of also selecting for AR. The clearest example of this is Tn*21* transposons (and their included class 1 integron), which carry both the *mer* operon, mediating resistance to inorganic mercury, and *qacE* genes, mediating resistance to QACs [83]. Co-selection of antibiotic resistance by the mercury released from dental fillings was demonstrated many years ago [84], as well as the effect of QACs on resistance, either via co-selection of *qacE* genes or cross-resistance [85]. Several other examples are listed in Table 2.

Another type of co-selection is cross-resistance, i.e., when single traits confer resistance to both antibiotic and non-antibiotic agents. Non-antibiotic compounds could select for several non-specific resistance mechanisms, resulting in increased AR. Fitting examples include triclosan, a disinfectant that was common in household products for many years and could even be found in the urine of humans using these products [86], and paraquat, a widely used herbicide. Triclosan is an efflux pump substrate that can also extrude antibiotics from bacterial cells [87] and induce the alarmone guanosine tetraphosphate, thereby mediating tolerance to several antibiotics [88]. In contrast, paraquat induces an antioxidant response, the *soxRS* regulon, which also confers AR [89]. Further examples are listed in Table 2. A number of non-antibiotic drugs have antibacterial activity at concentrations achieved in patients or environments that receive wastewater. Such compounds can potentially exert selective pressure for AR, from widely used ibuprofen [90] to the antiplatelet ticagrelor [91].

Much less obvious is the effect of agents that are not inherently noxious to bacterial cells, or that are not commonly present at toxic concentrations. Such agents exert subtle effects on bacterial physiology, resulting in modified responses to antibiotics. The herbicide glyphosate is a relevant example: by either increasing or diminishing the MICs of antibiotics, this compound can change the threshold of antibiotics needed to select for resistance in the environment [92,93].

Finally, many agents or conditions not only act as selective pressures or modifiers of antibiotic action themselves but also enhance the ability to surmount a defined selective pressure: agents that increase mutability or HGT can also increase the chance of acquiring a resistance determinant, and there is interplay between biofilm formation (which causes antibiotic persistence) and HGT, either as a cause or effect. Antibiotics, in addition to being the main selective pressure for AR, can induce HGT of AR genes, facilitating the acquisition of AR. Further examples are given in Table 2. These agents or conditions may play a secondary role in the selection and maintenance of AR.

**Table 2:** Agents that select/maintain and/or facilitate ^a^ the acquisition of antibiotic resistance traits.

Select/Maintain	Facilitate
*ANTIBIOTICS* ^b^	
Tigecycline can select for colistin resistance in hypervirulent *K. pneumoniae* [94].	Meropenem increases copy number of carbapenemase genes and promotes release and transfer of chromosome-integrated plasmids [95].
Tetracycline/sulfamethazine/penicillin supplement for swine selects for aminoglycoside-modifying enzymes [96].	**Antibiotics increase conjugation frequency**: - By RecA induction by ROS [97].
Allogenous selection, i.e., old antibiotics selecting for resistance to newer antibiotics of the same family [98].	- Of Vancomycin resistance in *E. faecalis* [99].
Stepwise exposure to amikacin can select for hyper-resistance (MICs 10–20 mg/mL) in *P. aeruginosa* [100].	- Cefotaxime increases expression of *tra* genes in *E. coli* [101].
Repeated exposure to ciprofloxacin selects for mutations in *gyrB* outside QRDR region, causing “hyperpersistence” [102].	- Tetracycline induces transfer of Tn*916* [103].
	- Sub-MIC antibiotics promote conjugative spread of AR genes [104].
	- Sub-MIC colistin promotes conjugation [105].
	- Enrofloxacin promotes conjugation [106].
	Tetracycline + Cu increases mutations to chloramphenicol and polymyxin B resistance in *E. coli* [107].
	Antibiotics induce mutagenesis, in turn increasing genetic diversity and “evolvability” [108].
	Macrolides induce biofilm formation in *S. aureus* [109].
	Aminoglycosides and fluoroquinolones induce transformability in *Streptococcus pneumoniae* [110].
*OTHER BIOCIDES*	
QACs induce and select changes that can also protect against antibiotics [111], increase AR genes in sewage sludge [112], and co-select for class 1 integrons in contaminated environments [113]; low-concentration QACs promote AR gene transfer by conjugation [114].	
Chlorophene, benzalkonium chloride, glutaraldehide, and chlorhexidine select for mutations in efflux proteins and porins conferring AR [115].	Surfactants SDS, cetyltrimethylammonium bromide, and benzalkonium chloride increase HGT by transformation [116].
Oral antiseptics (chlorhexidine, cetylpyridinium) increase AR in oral bacteria [117].	Chlorine promotes transformation [118].
Chlorhexidine exposure selects for azithromycin- and ciprofloxacin-resistant mutants in *N. gonorrhoeae* [119]; chlorhexidine–colistin cross-resistance in *K. pneumoniae* [120].	Prochloraz, a fungicide, promotes conjugation [121].
Glyphosate co-selects for AR [122] and induces imipenem resistance in *P. aeruginosa* [123]; glyphosate and dicamba modify ^b^ the effects of antibiotics [93]; glyphosate, glufosinate, and dicamba promote conjugation [124], and increase prevalence of AR genes and their transfer [125]; glyphosate affects persistence and tolerance without affecting antibiotic MICs [126].	
Dazomet (soil fumigant) increases the relative abundance of AR genes, incidence of mobile genetic elements, and conjugation [127].	
Cu, Zn, Cd, and Cr are used as feed additives in animal care; their resistance genes are linked to AR genes in conjugative plasmids [128].	Cu and Zn increase conjugation [129] (or inhibit conjugation by reducing the expression of conjugative genes [130]); Cu facilitates conjugative transfer of ICEs across bacterial genera [131].
	Paraquat confers tolerance in *P. aeruginosa* by inducing SOS and diminishing membrane permeability [132]; it induces *soxRS* in *E. coli*, decreasing antibiotic effects [89].
	Chloroxylenol promotes conjugation [133].
*OTHER DRUGS*	
Antivirals (zidovudine, dolutegravir, raltegravir) select for cross-resistance vs. trimethoprim, amoxicillin, tetracycline, and macrolides [134]	Carbamazepine promotes conjugation at environmentally relevant concentrations [135]. It is worth noting that carbamazepine is the most frequently detected drug in rivers worldwide [136].
Quetiapine activates *mar* and can select for *mar* mutations that protect against antibiotics [137].	
	Fluoxetine induces AR by ROS-mediated mutagenesis [138].
	Sertraline increases expression of AR and virulence genes [139].
	Paclitaxel enhances conjugation [140].
*MISCELLANEOUS*	
Deforestation increases AR gene presence in soil bacteria [141]; agricultural stressors (increasing temperature, loss of fertility, increased salinity) can increase AR in soil bacteria [142].	Environmental sources of oxidative stress can induce responses linked to AR [143].
Cosmetic components (e.g., parabens, triclocarban, triclosan) co-select and facilitate acquisition of AR [144].	Bisphenols promote conjugation [145].
Antifouling paint selects for efflux systems, conferring tetracycline and heavy metal resistance, and has lower taxonomic diversity in biofilms [146].	NO, byproduct of wastewater treatment, promotes conjugation [147] and induces *soxRS* in *E. coli*, which in turn results in AR [26].
	Sweeteners (saccharine, sucralose, aspartame, acesulfame) promote conjugation [148].
	Heavy atmospheric pollution decreases permeability in *E. coli* [149].
	Plant growth regulators (indolacetic acid, ethel, gibberellin) promote conjugation [150].
	Tilimycin, a toxin from *Klebsiella* spp., increases mutagenesis towards AR in gut microbiota [151].
	Phage infection and type VI secretion system attack and induce the *soxRS* regulon [152].

^a^. “select/maintain” refers to agents that exert direct selective pressure favoring a resistance trait, either a mutation or an HGT-acquired resistance gene, with co-selection and cross-resistance being the clearest examples. “Facilitate” refers to agents that promote mutagenesis or HGT, or that modify bacterial responses to antibiotics (e.g., by inducing tolerance), enabling the acquisition of resistance. ^b^. This lists examples other than the obvious selection of a resistance determinant by its corresponding antibiotic. Combined columns indicate that an agent has been reported both as a selective–maintenance pressure and as a facilitator; bordered cells group related agents. HGT: horizontal gene transfer; MIC: minimal inhibitory concentration; QAC: quaternary ammonium compound; QRDR: quinolone resistance-determining region; ROS: reactive oxygen species.

There are many different contexts where canonical and non-canonical selective pressures can interact. In clinical conditions, this may occur within a single patient treated with antibiotics and/or drugs exerting selective pressure, with mercury dental fillings, and triclosan-containing soap. Hospital floors or sinks are teeming with resistant bacteria and are routinely soaked with antibiotic solutions (e.g., the drops of injectable antibiotics coming out of a syringe or IV tubing when purging the air) and disinfectants. A wastewater treatment plant receives all of the above, and the resulting concoction is poured into water bodies. Culturable land receives resistant bacteria and antibiotics from manure, antibiotics used against plant diseases, and herbicides that modify bacterial responses to antibiotics. Other complex environmental issues, such as climate change, also seem to foster the emergence and spread of AR. Climate change may increase AR due to the bacterial physiological changes induced by temperature shifts [153] or its interaction with antibiotics [154], but much more likely because of increased infection rates, pollution dispersal, and disasters and their consequences (e.g., flooding, population displacement, damage to sanitation infrastructure) [155]. In fact, AR and climate change, both global problems with similar features, are also “intertwined challenges for public health” [156]. Finally, it is crucial to understand that the notion of “reserving some antibiotics for the treatment of human infections”, while leaving others to be used agriculturally, reflects a complete misunderstanding of how selection works and, once again, allows for financial interests to override any attempt at controlling the AR problem.

The many agents that can select/maintain or facilitate the acquisition of AR have consequences other than merely increasing AR prevalence. Often, AR genes are linked to determinants of an entirely different nature: virulence, mobility, stress responses, etc. When AR is selected by antibiotics or other agents listed in Table 2, it is also likely that linked traits are co-selected, resulting in dangerous combinations. The first report of AR and virulence traits residing in the same plasmid is more than 55 years old [157]; many more have been documented since. Biofilm formation is both a crucial virulence ability and an “enhancer” of HGT; the persistence of biofilms to antibiotics causes a vicious circle, making it difficult to separate cause and effect. Mobility traits are a very important component of the AR problem, and often co-occur with AR genes (Table 3). Antibiotics seem to modify the gene flux caused by HGT itself, potentially changing bacterial evolution in ways much more diverse than merely selecting for AR. This, aside from the fact that many “new” antibiotics are not actually new (see below), may be causing the apparent acceleration in the emergence of AR [158].

## 7. Non-Canonical Origins of Resistance Determinants

It is perhaps an exaggeration to state that mutations are still considered the canonical origin of resistance genes; after all, the acquisition of resistance being mobilized by conjugation was reported around 65 years ago (see below). Furthermore, the clear environmental origin of many clinically relevant resistance genes, such as the quinolone resistance *qnrA* gene or ESBL *bla*_CTX-M_ genes [169], leaves HGT as the only way that they could have reached current pathogens. Nevertheless, a recent review on interspecies interactions and their impact on AR focuses only on the emergence and spread of mutations, stating at the end that AR “can also spread in mixed populations via HGT. However, interspecies HGT is rare, as HGT mainly occurs between closely related strains” [170]. Of course, recent cases of AR have arisen due to mutations, but perhaps with the exception of fluoroquinolone-resistant *Campylobacter* spp. and enterobacteria, all other “priority pathogens” in the 2017 WHO list owe their resistance to horizontally acquired traits. (Similar to the WHO when listing the “priority pathogens”, we are leaving out *Mycobacterium tuberculosis*, where all AR known to date has been gained through mutations.) However, many notions around the current resistance crisis, such as AR being “caused” by the use of antibiotics, or that patients interrupting an antibiotic course “cause” resistance, seem to imply that AR emerges from susceptible bacteria via mutations [2]. And while, again, this is true in a reduced, isolated number of cases (and perhaps much more significantly for non-canonical resistance phenomena, such as tolerance or persistence), the bulk of AR in pathogens comes from HGT.

Two messages can be derived from this concept: (1) while it is understandable that papers dealing with mutations causing AR emphasize their findings as crucial to the AR crisis (e.g., Ref. [171]), perhaps it would be better to temper such claims; and (2) it is beneficial to avoid stating that the lack of resistance arising in a few bacterial cells exposed to a potential new antibiotic for a month supposedly predicts that bacteria “do not develop resistance to the drug” [172]. This is, first, because resistance is much more likely to arise because of HGT, especially for naturally occurring antibiotics (such as teixobactin, which also failed to select for resistant mutants [173]). Second, resistance can arise from a combination of mutations and HGT events with a likelihood much below the detection limits of any “risk assessment” trial, as the emergence of penicillin resistance in pneumococci showed [174]. In the absence of a dramatic selective pressure, “the process and outcome of HGT are often not amenable to experimental investigation” [175].

Resistance to two interesting drugs, fosfomycin and nitrofurantoin, does arise due to mutations, sometimes during treatment; however, acquired resistance rates remain very low. Mutations making *E. coli* resistant to these drugs have a significant fitness cost [176,177], making them unlikely to succeed in the absence of the respective antibiotic—and these are relatively little-used antibiotics with no known cross-resistance. Resistance to fluoroquinolones, on the other hand, is now very common, despite this drug family being among the latest in the antibiotic arsenal. High-level fluoroquinolone resistance (i.e., MICs above resistance breakpoints) mostly occurs through changes in the target enzymes gyrase and/or topoisomerase IV, especially in the so-called “quinolone resistance-determining regions” (QRDRs). Several stepwise mutations are necessary to gain the fully resistant phenotype, and such mutations are recessive, limiting the chances of the mutated genes being acquired via HGT [35]. Yet, fluoroquinolone resistance was acquired horizontally by the pandemic *E. coli* clone ST1193 by the transfer of 1 Mb of chromosomal DNA followed by several homologous recombination events that occurred about 15 years ago [178]. Hence, despite mutations being the actual origin of this resistance, HGT is still the main source of the clinically relevant AR problem. Beta-lactam resistance in *S. pneumoniae* has similar features: while mutations are the likely source of individual changes in PBPs resulting in decreased susceptibility to penicillins, current clinical isolates acquire these changes through transformation followed by recombination in a particular order to gain clinically relevant resistance [179].

Some mutations leading to AR have a rather unexpected effect on the fitness cost of carrying resistance plasmids: mutations causing nitrofurantoin, ciprofloxacin, and streptomycin resistance in *E. coli* mitigate the costs of bearing multi-resistance plasmids isolated from clinical strains [180]. Should this be proven in actual clinical environments, it could indicate that mutations can foster AR by providing better hosts for extrachromosomal elements.

In a recent analysis, a small number of mobilizable AR genes were proposed to have emerged mostly from pathogenic proteobacterial species that have been isolated from infections in humans or domesticated animals, where they “may experience severe antibiotic selection pressure” [181]. However, many of the supposed “pathogens” from where these genes originated seldom cause infection (e.g., *Shewanella algae*, *Kluyvera ascorbata*), and others are known opportunistic saprobes (e.g., *Acinetobacter baumannii*) with only limited hospital-related antibiotic exposure. Hence, it is much more likely that the genes originated in open environments where these bacterial species are much more abundant and then, indeed, to “encounter mobile genetic elements that have mobilized AR genes in the past”.

## 8. Non-Canonical Horizontal Gene Transfer

The first documented example of the transfer of resistance genes was observed between enteric bacteria within patients in 1960 [182]. Still, a fairly recent review aims to “demonstrate the human lower gastrointestinal tract as an environment in which HGT of resistance determinants occurs” [183]. HGT has remained in the realm of molecular microbiology, with most clinicians barely able to recite the HGT “trinity” (transformation, transduction, and conjugation); the profound impact of HGT on the evolution of AR is still neglected in medical literature and college microbiology texts. Furthermore, “the rates of horizontal transfer in clinical environments and the impacts of HGT on disease frequency remain unknown or speculative” [184]. Additionally, while the wrong notion of mutations being the main drivers of resistance is slowly subsiding with a stronger understanding of the role of HGT, the actual potential of HGT is often underestimated, as something that merely allows for resistance genes to travel from one strain to another. In fact, HGT has always fostered bacterial evolution, not only regarding AR, and keeps doing so at an accelerated pace.

**Figure 5:**
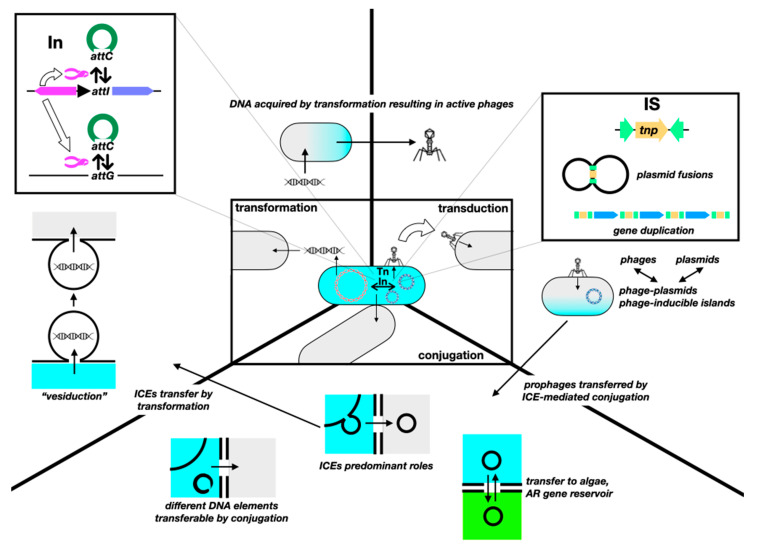
**HGT, old and new.** At the **center**, the canonical HGT “trinity” of transformation, transduction, and conjugation is represented in its most simplistic way; also, intracellular mobilization is carried out by transposons (Tn) and integrons (In) that exchange gene cassettes. **Radially**, some examples of recent concepts of HGT are shown: DNA acquired by transformation resulting in active phages [196], which combine transformation and transduction; prophages carrying AR and virulence genes being transferred by ICE-mediated conjugation [197], combining transduction and conjugation; and ICEs being transferred by transformation (e.g., Tn*916* in *S. pneumoniae* acquired by transformation and recombination [198]), combining conjugation and transformation. The figure also shows “vesiduction”, i.e., transformation of DNA carried by membrane vesicles; phage–plasmids carrying a number of AR genes (e.g., P1-like phage–plasmids carrying 48 different AR gene subtypes, including *mcr-1* and *tetX4*, mediating colistin and tigecycline resistance, respectively [199]) and exchanging genes with plasmids and phages, even turning into plain phages or plasmids [200]; and phage-inducible chromosomal islands from Gram-positive cocci [201]. The predominant role of ICEs in mobilizing AR genes, as well as other elements capable of conjugative mobilization (including the whole chromosome of mycoplasmas and circular elements of *Campylobacter*). Interkingdom conjugation, well known since the reports of conjugation between bacteria and yeast, as well as *Agrobacterium tumefaciens* plant infections [195], is now making algae potential reservoirs of AR genes [202]. (**Top left**) A typical integron, with an integrase gene (magenta), a promoter (black), an attachment site (*attI*), and an already integrated cassette (blue); integrases (magenta pliers) catalyze the insertion or excision of gene cassettes (green) by recombining *att* sites. It was recently reported [203] that integrases can also insert/excise gene cassettes at other similar attachment sites in the genome (*attG*), where the cassette can express if a promoter is near, or can alter chromosomal genes. (**Top right**), the increasing roles of insertion sequences (ISs) in rearranging and mobilizing AR genes.

The sole variety of known mobile genetic elements has grown inexorably since the discovery of HGT. In early notions of intercellular gene mobilization, naked DNA from dead bacteria was capable of transforming; phages could mobilize genes by either generalized or specialized transduction; and conjugative plasmids could mobilize themselves or also chromosomal fragments if inserted into the chromosome. Intracellularly, transposons could make genes “jump” between replicons. Then came the “mobilizable” plasmids, capable of being transferred but not encoding the whole conjugative machinery; replicons with a dual plasmid–phage nature; conjugative transposons, capable of “jumping” between replicons and cells; and integrons, capable of capturing, shuffling, and enhancing the expression of gene cassettes, frequently containing AR genes [185]. Now, we have insertion sequences capable of mobilizing genes in ways different from merely assembling a composite transposon; MITEs and TIMEs, which are elements capable of transposon-like mobilization but only in the presence of compatible full transposons; phage satellites, phage-inducible chromosomal islands, and phage-inducible minimalist islands that hijack the machinery of phages for their own dissemination [186]; integrative conjugative elements (ICEs, including the former conjugative transposons) of many kinds; and “resistance islands”, such as the Staphylococcal Cassette Chromosome *SCCmec* responsible for “methicillin resistance” [187]. Peculiar “gene transfer agents”, particles that resemble phages but cannot self-propagate, and that mobilize random pieces of the host’s DNA, have been described in a few bacterial genera (e.g., *Ruegeria*, *Brachyspira*, *Desulfovibrio*) of unlikely clinical relevance [188]. Furthermore, the actual boundaries of the three HGT mechanisms became blurred with the discovery of a fourth, “vesiduction”, i.e., transformation via membrane vesicles [189,190]. HGT is an evolving trait, with continuous changes in fitness costs, defense systems, recipient availability, plasmid exclusion, etc. [191]. For example, while the overall prevalence of the mobile colistin resistance gene *mcr-1* in *E. coli* decreased between 2016 and 2019, it is now linked to stabler genetic structures and to additional resistance and virulence genes, moving towards extraintestinal pathogenic strains [192]. This evolution affects not only the bacterial host but also plasmids, and it occurs within patients [193]. It is important to understand the dynamic of this evolution, particularly in the presence of the selective pressures discussed above. There are plenty of reviews on HGT (e.g., Refs. [194,195]). A graphic view of the expansion of HGT mechanisms is shown in Figure 5.

Genes mobilize intracellularly, via integrons (In) and gene cassettes, insertion sequences (ISs) and transposons (Tn), in addition to homologous recombination. ISs are often underestimated as transposons “without cargo”, hence unable to mediate the mobilization of AR genes unless assembling a composite Tn. However, they seem to play a significant role in HGT and AR. For instance, streptococcal IS*1216E* can mediate the integration of different ICEs and then be conjugatively transferred along with the ICE [204]; IS*26* mediates increased copy numbers of *bla*_CTX-M-65_, resulting in higher MICs without a fitness cost in *E. coli* [205]; IS*26* also frequently flanks *bla*_NDM_, suggesting an important role in the dispersion of this resistance gene [206]. There seems to be a network of gene transfers, mediated by ISs, and mobilizing genes between conjugative plasmids in distantly related pathogenic bacteria [207]. ISs can also play a role in the emergence of AR in unsuspected ways: IS*1* causes deletion of chromosomal *nfsB*, resulting in nitrofurantoin hetero-resistance [208]. A somewhat similar role was proposed for IS*1* in the emergence of resistance towards nalidixic acid in an old report [209], and for IS*Kpn72* inserted into gene *mgrB* of *K. pneumoniae*, causing colistin resistance [210]. Also, IS*ba* elements, containing a strong promoter pointing outwards, are responsible for the overexpression of AR genes, especially those conferring resistance to carbapenems [211] (this phenomenon was first described for non-AR genes in non-clinically relevant *Rhizobium*, nearly 35 years ago [212]).

Intercellular mobilization is mediated by the aforementioned “trinity”. Conjugation has long been considered the main route for the HGT of AR genes, as there are purported limitations for either transformation or transduction (e.g., low half-life of free DNA for the former; narrow host spectrum for the latter [59]). However, both transformation and transduction may easily rival conjugation as a means for AR gene spread. With the millions of copies per milliliter of extracellular DNA encoding carbapenemases being dumped into wastewater [213]; the fact that fragmented (≥20 bp) and damaged DNA can be acquired by transformation [214]; that transformation can occur in unexpected places, such as the phylloplane of edible vegetables [215]; and AR genes being routinely found in phages (even towards the “last resort” antibiotic colistin [216])—from the fecal “phageome” of healthy humans [217], food samples [218] (up to the point that the liver of farm chickens can be considered as a reservoir of AR genes [219]). Furthermore, genomic islands shared by distantly related bacteria were likely mobilized between them by phages [220]. While transformation is often conceived as the almost passive uptake of free DNA, transformation competence is induced by different forms of stress, most likely to achieve genetic diversification and survive such stress [221].

Conjugation is often thought to be driven by, and involving only, conjugative plasmids, which should exert a significant fitness cost in the absence of selective pressure because of their large size and the expensive nature of the conjugative machinery. However, this purported fitness cost is seldom found in nature; there are many reasons for this persistence [222]. For instance, compensatory mutations emerge rather quickly to restore fitness after the acquisition of plasmids [223]. On the other hand, resistance acquired by HGT has lower fitness costs than that gained by mutations, “which can contribute to the observed dominance of horizontally transferred genes in the current AMR epidemic” [224]. Conjugation also enables the persistence of AR even in the absence of antibiotics; hence, “reducing antibiotic use alone is likely insufficient for reversing resistance” [225]. Furthermore, many mobile elements other than the “conjugative plasmid” do mobilize conjugatively. “Hitchhiking” mobilizable plasmids carry a significant proportion of AR and virulence genes in *K. pneumoniae*—whose transfer and stability are not affected by CRISPR-Cas systems ([226]—which may also act as “back-up” copies of AR genes, due to their higher copy number, and enable evolution by the coexistence of mutated and non-mutated versions [227]). Additionally, the role of ICEs in mobilizing AR genes is increasingly recognized [228]. Carrying plasmids could also have unexpected consequences; bearing conjugative IncF plasmids with *bla*_CTX-M_ genes increases the frequency of mutations 10–1000-fold in topoisomerase genes, resulting in fluoroquinolone resistance [229]. Some pathogens conjugate using entirely different elements: *Mycoplasma* spp. transfer chromosomes differently from the Hfr/*oriT* model [230], and there are some circular intermediates in the chromosome and plasmids of *Campylobacter coli* that can insert, excise, and be horizontally transferred by conjugation, carrying different AR genes [231].

Of course, mobile elements impact the genome of their hosts: genomes associated with phages and plasmids are significantly larger than those that are not, and genomes with CRISPR systems, which limit the acquisition of phages and plasmids, are significantly smaller than those without [232]. The evolution of plasmids allows them to overcome transfer and segregation barriers: by searching sequence databases, it was hypothesized that plasmids now tend to carry multiple origin-of-transfer (*oriT*) sites, which could extend their mobility to form “robust plasmid transfer networks” [233]. Also, multi-replicon plasmids, such as those from *Klebsiella*, are able to circumvent incompatibility limitations and spread easily [234], a phenomenon first reported in 1991 [235]. And while it was hypothesized that plasmids with post-segregational killing (psk) abilities may not coexist with non-psk plasmids within the same bacterial population, coexistence appears rather common and is made possible by spatial structures the kind are found in biofilms [236].

Some particularly dangerous clinical consequences of this unbridled and evolving HGT are listed in Table 4.

## 9. Non-Canonical Dispersion of AR

AR is mostly searched for in isolates from infected patients rather than clinical environments, so the recent finding of resistant *P. aeruginosa* strains in hospital sink drains made it to a high-impact journal [250]. This is perhaps the consequence of the CDC “advocating the discontinuation of routine environmental culturing” in hospitals in 1970 [251]. Only recently has the investigation of AR outside clinical settings finally been considered highly relevant as it should have been for a while. Just to exemplify this, in 2023, the United Nations, “too litle, too late” as usual, published a report on the “environmental dimensions” of resistance alerting that resistance genes come and go from the environment at rates exceeding those in clinical settings [252]. However, when facing an infection caused by resistant bacteria, most physicians believe it was acquired either by resistance emerging due to previous antibiotic treatments, as discussed before, or by contagion, including the typical failure to wash hands in healthcare facilities. Little thought is given to the fact that resistant pathogens and/or resistance genes (that can be transferred to pathogens afterwards) are acquired in many other ways.

Foodstuff is a common source of AR. With most antibiotics produced worldwide being used for agricultural purposes, it is no surprise that most foods of animal and vegetal origin contain resistant bacteria [59]. For instance, in the US alone, 20–52% of broiler operations use around 3.3 million kg of antibiotics to produce 9 billion broilers and 14 million tons of litter annually [253]. Chicken meat carries bacterial pathogens so often that 40–50% of human campylobacteriosis in the EU and US came from poultry (and 30% of salmonellosis in African and eastern Mediterranean regions [254]). Workers in direct contact with medicated animals (e.g., farmers, veterinarians, abattoir workers) are often colonized by resistant bacteria, representing “an entryway [for them] into the community” [255]. Airborne bacteria within and near farms carry more AR and virulence genes than those isolated from hospital air samples [256]. Arthropods carry AR bacteria from livestock units into surrounding areas [257] (a report from Germany shows that flies carrying *bla*_CTX-M-1_ and fluoroquinolone resistance genes from pig farms can be found 2 km away into urban areas [258]). Livestock production is a major source of AR genes in the soil [259]. Manure, used to fertilize crops, carries antibiotics and resistant bacteria to soils, water and, of course, the fertilized crops. Fresh produce often carries AR bacteria or genes, e.g., 95% of samples of a recent Swiss study contained AR genes, in many cases along with antiseptic resistance determinants in mobile elements [260]. Cultured fish and seafood also receive antibiotics: more than 10,000 tons per year (predicted to grow to 13,600 tons in 2030 [261]). It is important to emphasize that the risk posed by resistant bacteria in foodstuff is not limited to enteric infections. Just as an example, uropathogenic *E. coli*, the causative agent of one of the most common community-acquired infections, likely comes from poultry [262]. However, even AR genes carried by innocuous bacteria have an enormous risk of being transferred horizontally to pathogens in the kitchen or the guts of people handling or eating contaminated food.

Perhaps the most puzzling aspect of the continuous use of antibiotics for growth promotion is that, despite some authors considering that eliminating it “will result in higher meat prices and an inevitable increase in poverty through lack of income” [263], the impact of a ban would be a reduction of 0.31–0.47% in output, and an increase of 0.73–0.77% in wholesale price [264]. Given this cost–benefit balance, it is simply immoral to keep using antibiotics for this purpose, yet it is expected to grow further [265].

As a consequence of both the clinical and the agricultural use of antibiotics, AR bacteria and genes can now be found almost everywhere. The presence of AR in the environment has been extensively reviewed before (e.g., Refs. [59,266]); here, we would only like to highlight two major issues (wastewater and wildlife) and list (Table 5) some examples of AR being reported in odd or unexpected places, as a reminder of the many ways AR can spread.

Wastewater (WW) is a unique mixture that contains antibiotics, biocides, and many other previously listed agents that select/maintain and/or facilitate the acquisition of AR (e.g., Refs. [294,295]), along with pathogenic and commensal bacteria, from humans and animals, many of them carrying AR, virulence, and/or mobility determinants. Of particular concern is the WW of hospitals, where particularly dangerous multi-resistant organisms can be found (e.g., Ref. [61], although a report indicates that there is more AR in common urban wastewater [296]). Pharmaceutical companies [297,298] also dump antibiotics and active derivatives, along with other drugs that can exert selective effects. This mixture can be directly discharged into water bodies, especially in non-developed countries, or treated in WW treatment plants (WWTPs) to reduce the amount of toxic chemicals and bacteria before being discharged. Even before treatment, the mixture can select for AR, as reported in hospital effluents [299]. While WWTPs can significantly reduce the amount of antibiotics released into the environment [47], they act as a sort of reactor for the shuffling and concentration of AR genes and bacteria (e.g., Ref. [300]), and although some treatment processes can efficiently remove AR [301], they are seldom used, especially in poor countries, whileothers can actually select for AR [302]. By putting together pathogens, commensals and environmental bacteria, AR and mobility genes, and selective pressures favoring AR, WW is an ideal environment for the emergence of mobile AR genes that can then be spread into different bacteria, including pathogens [303,304]. AR genes that enter the environment, mostly onboard bacteria from humans and animals, are then transferred horizontally, as they are commonly linked to mobile elements [305]. The impact of treatment on some relevant AR genes is discussed in [306]. Some final snippets on this issue include the following:

- Biosolids from WWTP, often used as fertilizers, contain a significant load of AR genes, in many cases within conjugative plasmids [307].
- Biofilms exposed to even low-concentration antibiotics in aquatic environments, such as water bodies and WWTPs, serve as hotspots for all sorts of mutations and HGT events, becoming reservoirs of AR bacteria and genes [308].
- Between 860 and 14,500 tons of extracellular DNA are discharged into water bodies per year, some containing around 10^7^ copies per milliliter of genes such as the carbapenemase gene *bla*_NDM-1_ [213]. The risk of such AR genes being acquired by transformation-competent bacteria, even if small, is inevitable.
- Fish inhabiting water bodies that receive wastewater often carry AR bacteria. For instance, nearly 21% of fish from rivers receiving inputs from WWTPs in Ohio, US, carry cephalosporin-resistant bacteria, and 80–88% of intestinal samples contain carbapenemase genes [309].
- To reduce the concentrations of macrolides and fluoroquinolones dumped into the River Thames below a “putative resistance-selecting concentration”, it would take a 77% reduction in macrolide prescriptions and an 85% reduction in fluoroquinolone prescriptions [310].

AR has been found in bacteria isolated from different forms of wildlife for more than 40 years [311]. Even animals living in extreme environments, such as arctic reindeer, carry AR bacteria [312]. Resistance to the oldest drugs, such as ampicillin, sulfonamides, and tetracycline, is rather common, while resistance towards fluoroquinolones and third-generation cephalosporins is lower. (Fortunately, resistance to carbapenems and polymyxins is almost non-existent, but genes conferring resistance to novel antibiotics, such as tigecycline, were found in the fecal microbiome of migratory birds [313].) In some cases, the prevalence of resistance is linked to the closeness of sampled animals to human settlements; in most cases, attention has been given primarily to *E. coli*, although other bacterial species can yield unsuspected results [59]. Besides clearly indicating the reach of the pollution caused by the human release of antibiotics and AR bacteria and genes, little is known about the impact of having AR in wildlife, on human health or otherwise. Particularly worrisome is the carriage of AR by birds [314], especially migratory ones, since they can mobilize such resistant microorganisms across wide areas and country borders, limiting the impact of local measures to diminish antibiotic usage [315].

The speed at which AR genes can disperse is astonishing. Take genes encoding New Delhi Metallo-beta-lactamase (NDM)-type enzymes: first reported in 2009 (*bla*_NDM-1_ [316]), there are currently at least 25 variants reported worldwide [317]. Its fifth variant, *bla*_NDM-5_, first reported in England in 2011 [318], was recently found in an *E. coli* IncX3 plasmid from a farm dog in Lebanon [319], as well as in *Citrobacter sedlakii* IncX3 plasmids from outdoor aerosols in a WWTP [320], in chicken and pig farms [321], in hospital wastewater in China [249], and in a patient in Japan [322]. It was even found in *E. coli* IncF plasmids from wastewater of Mexico City (without even being reported by local clinical sources [323]), and in *E. coli* isolates from hospitalized patients in England [324], Italy [325], and China [326]. The fourth sulfonamide resistance gene, *sul4*, is now arising as a potential clinical problem. First detected by metagenomics in a river sediment in 2017 and then in other environments, especially in marine bacteria [327], it has now been detected in *Salmonella enterica* [328].

Contrasting with the vast evidence of AR being all around is the scarcity of clear links between environmental and clinical AR bacteria and genes, to the point of some studies suggesting that “the vast majority of clinical cases origin[ates] from other humans”. Sample bias, dismissing the reservoirs established in commensals but capable of being transferred horizontally to pathogens, and low-resolution techniques that could miss the actual origin of AR are among the possible causes of this discrepancy [329].

## 10. Non-Canonical Consequences of Resistance

As the main result of resistance is supposedly the clinical failure of antibiotic treatments, almost all measurements of the consequences hinge on it, e.g., the 50 million deaths and USD 10 trillion that treatment failure will supposedly cost by 2050 [330]. But what about the effects of antibiotics and AR bacteria on all other organisms and environments routinely receiving them? Arguably, the changes in the microbiota of food animals that receive antibiotics [96] are of no concern—they are sentenced to death anyway. Nevertheless, the effectivity of antibiotics in such animals is only expected to decrease as resistance grows—and it does keep growing, especially in low- and middle-income countries [331]. Also, just as the use of antibiotics in animals has a direct impact on AR in human pathogens, antibiotic consumption by humans affects AR in food-producing animals [332]. Animals that spend their lives in the wild also receive antibiotics, either directly or indirectly. Beekeeping, for instance, uses antibiotics, and the welfare of bees is not only of interest because of honey production but also (or rather mostly) for their invaluable pollination activity. Antibiotic administration to honeybees alters their microbiota in ways that are transferred to following generations, and this dysbiosis affects the health of the animals [333]. Aquatic animals that live in water bodies used for aquaculture are also exposed to high doses of the drugs intended for farmed fish.

An issue almost completely neglected around the environmental release of antibiotics and resistant bacteria is its impact on soil and aquatic microbiotas. We do know that resistant bacteria can be isolated from soil and water receiving WW, manure, etc., but we know close to nothing about the effects of these “newcomers” on the microbial ecology, and the interactions of affected microbiotas with plants and animals in the wild. As discussed before, AR can be detected in wildlife all over the planet; even animals far from any human settlements carry AR bacteria in a way that mimics human antibiotic use and production [334]. If antibiotics fed to farmed animals modify their microbiotas to the point of changing their metabolism [96], could the same effect be happening to wild animals exposed to the antibiotics dumped into their environments? Soil and plants could also be affected by the large-scale release of antibiotics and AR bacteria and genes. Sulfadiazine in soils affects the amount and diversity of ammonia-oxidizing archaea and bacteria, as well as the soil nitrification rate [335]. The massive release of resistant bacteria carrying conjugative plasmids into the biosphere can have unexpected consequences: *Geobacter sulfurreducens*, which contributes to the geochemical iron cycle and other electrochemical systems, can have its extracellular electron transfer inhibited by the burden of conjugative plasmids [336]. Of course, the use of antibiotics against plant pathogens also results in the selection of resistance: *Xanthomonas arboricola*, which causes bacterial spot in peaches, now carries tetracycline and streptomycin resistance genes [337]. With climate change also affecting the composition and “services” provided by the soil microbiotas [338,339], it would be difficult to assess the combined impact of both climate change and AR spillover on the microbes of the soil.

## 11. Non-Canonical Approaches to Fight Resistant Infections

Research and development (R&D) of new antibiotics is still the main route in the fight against infections caused by resistant bacteria. Several initiatives have come and go, attempting to “jump-(re)start” antibiotic R&D by large, transnational pharmaceutical companies (i.e., *Big Pharma*): “10x’20” intended to have ten new antibiotics by 2020 [340], which failed; then, “5 by 25” [341] reduced the expectation but had similar results. There are two main obstacles for these strategies, one biological and the other financial. On the biological side, it is likely that the antibiotic boom of the 1950s–1990s was only caused by the “low-hanging fruit” nature of bacterial targets and antibiotic molecules [2]. Financially, there is stark contrast between funding, such as the Combating Antibiotic-Resistant bacteria Biopharmaceutical Accelerator and its USD 500 million to support research [342], and the USD 2.3 billion it costs to develop a new drug on average. Governmental plans to “jump-(re)start” antibiotic R&D consist mostly of giving away public monies to the same companies that are at the root of the problem. This happened to the “Generating Antibiotic Incentives Now” (GAIN) act in the US, which was only used for rehashed drugs for non-critical indications [343], and seems to be the same for the UK’s “subscription model” [344]. As always, it is all about “incentives” (or rather bribes) for *Big Pharma*, ignoring that, in addition to carrots, there are few sticks available [345].

Importantly, “new antibiotics” actually mean “antibiotics with entirely new mechanisms and/or scaffolds”, and not merely minor chemical modifications to old drugs, as resistance can likely also emerge from minor modifications to old resistance mechanisms (e.g., Ref. [346]). Yet, most “new” drugs, already available or still in the R&D pipeline, are mere “rehashed” ones: fourth-generation quinolones (e.g., delafloxacin, nadifloxacin, lascufloxacin) and macrolides (e.g., solithromycin); third-generation beta-lactam inhibitors (e.g., vaborbactam, relebactam), aminoglycosides (e.g., plazomicin), and tetracyclines (e.g., eravacycline, omadacycline); and a variety of derivatives of other old drugs (e.g., “siderophore” cephalosporin cefiderocol, trimethoprim derivative iclaprim, linezolid derivative contezolid, penicillin derivative sulopenem) [347,348]. Entirely new antibiotics are so rare that they are now featured in top journals (e.g., teixobactin [173] and zosurabalpin [349]). As with most other drugs originating from pharmaceutical companies, their actual clinical benefit is dramatically low: 2.6% of drugs coming from *Big Pharma* provide such a benefit [350].

Among the “non-antibiotic” options explored to prevent bacterial infections, there are a number of vaccines, immunostimulatory agents, and probiotics, and to treat infections, some antibodies, bacteriophages, and derivatives, and antimicrobial peptides [351]. One of the most hyped options for the “post-antibiotic era” is rehashing the very old notion of phage therapy. While it is certainly interesting, the therapeutic use of phages is following many of the errors made with antibiotics, with the risk of rapidly losing their efficacy and/or causing adverse effects both clinically and environmentally [352,353,354]. In a rather lengthy minireview [355], a Phage Taskforce answers relevant questions regarding the future avenues of phage therapy research. Yet, little room is devoted to crucial questions, and their “suggestions” are almost always based on “known knowns”, such as looking for known sequences of AR genes, toxins, and integrases before using a phage for therapy. But there is no discussion of the dangers of phage resistance or phage-mediated HGT; the very questionable use of phages in agriculture, already undergoing further exploration [356,357] (although hopefully hindered by regulatory issues [358]); the genetic manipulation of phages to extend their spectrum (or phage “cocktails”, e.g., Ref. [359]); and the sinister combination of both [360]. As such, it appears we did indeed not learn the lessons of the antibiotic era.

Aside from the now almost canonical options to fight bacterial infections in the “post-antibiotic” era, several less-explored avenues could offer useful tools [361,362], although many have such narrow spectra that they are unlikely to attract interest from the crucial financial point of view. These include drug repurposing, nanoparticles, antimicrobial peptides, photosensitizers, etc. Targeting virulence to combat infections is a long-standing notion (e.g., Ref. [363]), but no drug currently in use has come from this research. Additionally, some options that have received less attention are listed in Table 6.

Vaccines, by preventing bacterial infections, reduce the use of antibiotics and could prevent the further emergence or spread of AR [381,382]. Paradoxically, many people consider antibiotics as safe and vaccines as hazardous [383], and disinformation is driving a decline in vaccination—and an increase in vaccine-preventable infections [384]. Therefore, it is unlikely that advances in vaccine R&D would be enough to affect the growth of AR or perhaps even to attract financial support.

Antibiotic-saving strategies need to be seriously explored. Urinary tract infections (UTIs) are the most common outpatient infections [385], and antibiotics are recommended as first-line treatment; hence, UTIs are among the main causes of antibiotic prescription [386]. This is despite evidence that two-thirds of uncomplicated lower UTIs resolve without antibiotics [387], with symptomatic treatment being enough to manage most cases. Some approaches specifically designed against UTIs could prove useful [388], although they have very limited power against other infections.

Along with new antibiotics and new ways to prevent or treat bacterial infections, it could be useful to reduce the burden of AR itself. This would include a reduction in selective pressures and transmission, and a restoration of susceptible populations [389]. However, all of this is much easier said than done.

While the list seems promising, in our opinion, we are very far from being able to declare the crisis averted, but some do [390].

## 12. Non-Canonical Influences upon Resistance Prevalence: The Societal Side

While it is clear from a biological point of view that AR is selected and maintained by the pressure exerted by antibiotics and other xenobiotics, the prevalence of resistance among bacterial pathogens in clinical settings seems to be influenced by other, non-biological factors. The WHO, for instance, lists some issues that accelerate AR prevalence, aside from antibiotic consumption: lack of access to clean water, sanitation, and hygiene for humans and animals; poor prevention and control of infection and disease in healthcare facilities and farms; poor access to quality, affordable medicines, vaccines, and diagnostics; and lack of awareness, knowledge, and legislation and/or enforcement [391]. “No access to clean water, open rather than closed sewage systems, variation in healthcare infection-control practices, inadequate provision of antimicrobials and diagnostics, farming systems with suboptimum regulation of antimicrobials, and high population densities” [392] are all issues that may exacerbate the prevalence of AR. The effect of systemic factors, such as political, economic, and societal influence; healthcare management; and policy and regulations, has started to be recognized as a driver in the process that modifies AR, but is still overshadowed in the literature. These factors have, in some cases, shown larger and more direct impacts than antibiotic misuse [393]. It can be difficult to understand how such issues can influence AR prevalence and spread; simply put, they affect the pathways in which AR spreads from reservoirs to the environment, so that once resistance is acquired, socioeconomic factors exacerbate its prevalence [394]. This can be seen clearly in countries with poor sanitation, poor waste management, and/or poor governance, which have high AR prevalence but comparatively low antibiotic usage [395]. On a global scale, AR prevalence is higher in low- and middle-income countries (LMICs) than high-income countries (HICs) [396]. This also means that the burden on the economy, society, and public health is also higher in LMICs [397]. These differences are often attributed to antibiotic use in each country, which is well accepted in the literature [75]; however, the higher use of antibiotics in some HICs is not accompanied by higher prevalence rates. This lack of correlation was shown by Hou et al. [398], with a high prevalence of AR in many LMICs and low prevalence in many HICs; inversely, HICs have a higher rate of antibiotic use than LMICs. It is important to state here that all these studies focus on AR prevalence among clinical isolates from infected patients. A metagenomic analysis of healthy individuals not taking antibiotics from ten countries (mostly HICs, but also including China, Israel, Kazakhstan, and Madagascar) showed a significant correlation between local antibiotic usage and AR gene abundance and diversity (except for China [399]). This is perhaps a reminder that the biological phenomenon of AR responds mostly to selective pressure, but the prevalence of AR in pathogens causing infections is affected by societal factors as well. Furthermore, AR is often measured in ways that do not represent the actual prevalence (e.g., bias from mostly sampling patients already treated), and selection is only half of the driving force behind AR (and the one receiving the most attention), with transmission being the other, neglected, half [28]. In any case, more than simple “globalization”, increased migration from LMICs to other LMICs, as is often the case of refugees, and to HICs, in the case of migrant workers, amounts to tens or hundreds of millions of people, with AR carriage or infection around 25% [400], making the problem one without borders and impervious to local measures.

There is strong evidence that socioeconomic factors shape health outcomes [401]. The social determinants of health (SDH) model by Dahlgren and Whitehead [402] divides the factors that threaten, promote, or protect health into three layers: general socioeconomic, cultural, and environmental conditions; social and community networks; and individual lifestyle factors. Some studies suggest that 80% of the determinants of health outcomes can be attributed to SDH, while medical care accounts for only 20% [403]. Although seldom looked at in this way, treatment failures due to AR fit the definition of “health outcome” that are influenced by SDH. The evolving but still scarce knowledge on socioeconomic drivers of AR could result in a new approach to the problem and change priorities in its mitigation [404,405]; this is especially relevant in LMICs, as discussed above.

In a recent systematic literature search [406] designed to find the socioeconomic factors impacting AR prevalence that included 13 articles, five factors were found to have an association with AR (Table 7): income, governance, health expenditure, infrastructure, and access to healthcare. Some of the papers compared the aforementioned socioeconomic factors to the use of antibiotics regarding their impact on AR prevalence. The conclusions included the following: (a) antibiotic consumption is overshadowed by specific socioeconomic factors, with governance being “potentially as important a determinant of AR as is antibiotic usage in people” [407]; (b) corruption is the main socioeconomic factor behind AR prevalence, so that “once the control of corruption indicator is included as an additional explanatory variable, 63% of the total variation in AR is explained by the regression, while only 28% of the total variation in AR is attributable to variation in antibiotic usage in people” [407]; (c) antibiotic consumption is not strongly associated with AR levels [408]; (d) antibiotic usage was positively associated with AR in only one out of three pathogens analyzed [409]; (e) the use of fluoroquinolones in humans was positively associated with the prevalence of fluoroquinolone-resistant *E. coli* in HICs, but not in MICs [410]; and (f) the positive effect of ambulatory consumption of antibiotics upon AR prevalence decreased in importance in a multivariate analysis [411] and usage “only explained a minor part of the occurrence of AR across the world” [412]. As a worthy comparison, the prevalence of AR in isolates from livestock also does not correlate with antimicrobial usage alone: usage history, mobile populations, environmental reservoirs, etc., also contribute to the persistence of resistant bacteria in animals [413].

The incorporation of socioeconomic (and even psychological and cultural [421]) factors into the AR equation has made some authors (e.g., Ref. [422]) consider AR as a “wicked” problem, a designation introduced in 1973 to address planning and governing of societal problems so complex that standard problem-solving mechanisms cannot be used, as the wicked problem is too complex and multidimensional [423]. Going further, AR might even be considered a “super-wicked” problem, as (a) time for finding a solution is running out, (b) those seeking to solve the problem are part of the cause, (c) central authorities to address the problem are either weak or non-existent, and (d) policy responses discount the future irrationally [424]. Defining a societal challenge as a super-wicked problem means that policy must take into account interrelated fields and conflicting goals. Furthermore, AR is a transboundary problem, both cross-border and multisectoral; it is a crisis of modernity, very much like climate change and the obesity epidemic, but it is a crisis without advocacy, with different impacts across countries and demanding different solutions [425]. This is the actual size of the AR problem, and where most of the strategies devised to control it fall short worldwide.

A final consideration regarding the economic issues behind AR: it is crucial to realize and to remember that the original cause of this problem was simply greed. As discussed before [426], this is “a profit-driven plague” that was caused by the financial interests behind antibiotic abuse, especially for agricultural purposes. Unless we stop letting these interests rule over public health, all this will happen again. 

## 13. Concluding Remarks

All the evidence and discussion above converge into a single, old concept: the resistance of resistance [427]. With knowledge gaps regarding what antibiotics do and what resistance is, and the wide variety of selective pressures, mechanisms, dispersion means, and non-biological influences, it is clear that the ubiquitous notion of “controlling” AR, as synonymous with “reducing” rather than “stabilizing”, through simply diminishing antibiotic use is not going to happen. “Reducing antibiotic use alone is unlikely to solve the AMR problem, and more interventions are needed to increase governance efficiency at global level” [415]. Small-scale antibiotic stewardship interventions, while effective in reducing the prescription of antibiotics, only have a small, brief effect upon AR, with an “overall trend remain[ing] on an upward trajectory” [428]. The European experience taught us as much: while the use of antibiotics diminished from 494–534 DDD per thousand inhabitants in 2008–2009 to 256 in 2018, the resistance average increased from 20–22 to 24.5 in the same time period [429]. At the gene level, there are even more dramatic contradictions: for instance, the carriage of genes encoding the most common N-acetyltransferases (aminoglycoside-modifying enzymes) is 1.5% in France and 18.1% in Austria, while the use of aminoglycosides is 10 times higher in the former than in the latter [430]. Furthermore, AR is a global problem that is not confined by country borders. “Any resistant microorganism (and its resistance genes) could be distributed worldwide [hence it] is a pandemic that requires Global Health solutions” [431]. However, we will be unable to devise such solutions until we fully understand the problem.

## Figures and Tables

**Table 1:** Non-canonical mechanisms of antibiotic resistance.

Mechanism Basis	Effect
Changes in cell size/shape	- Reduces drug influx or increases surface-to-volume ratio, diluting antibiotics acting upon cell wall or membrane [64]. - Cell wall deficiency (L-forms, spheroplasts) caused by beta-lactams is associated with persistence [65]. - Switching to and from L-forms is linked to recurrent urinary infections during treatment with cell wall inhibitors [66].
Membrane vesicles	- Quench polymyxins, rendering formerly inhibitory concentrations ineffective. - Beta-lactamases in vesicles protect the producing organisms as well as other surrounding, susceptible bacteria [67]. - Protect producing bacteria by exporting damaged proteins or membranes. - Contribute to biofilm formation after antibiotic exposure [68].
Metabolism	- Metabolic enzymes can protect against antibiotics: bile salt hydrolase degrades beta-lactams and confers resistance [69]. - Plasmids carrying metabolism-related genes result in higher growth and reduced killing by antibiotics [70]. - Mutations in carbon and energy metabolism genes lead to lower basal respiration, less antibiotic induction of Krebs cycle activity, and diminished toxicity of streptomycin, ciprofloxacin, and carbenicillin [71]. - Stress responses, non-specific efflux pumps, quorum sensing, and metabolic regulatory networks underlie bacterial tolerance and persistence in vivo [72]. - Pyoverdine, a siderophore from *P. aeruginosa*, causes cefiderocol tolerance, and high producers can cross-protect neighboring bacteria from the same drug [73].
Oxidative stress responses	- As ROS contribute to the action of some antibiotics, oxidative stress responses may counteract their action (see above). - The *marRAB* regulon and overlapping *soxRS* regulon of *E. coli* both confer multi-resistance [16]. - An “anticipatory gene regulation” that occurred when *E. coli* was grown in alternating media containing rhamnose and paraquat (a superoxide-generating agent) partially induced the *soxRS* regulon after exposure to rhamnose [74]; bacteria may encounter such cycling environments during infections.

**Table 3:** Examples of phenotypes co-selected with AR by antibiotics or other selective pressures.

Phenotype	Example
*VIRULENCE*	
Virulence determinants	- Hypervirulence and carbapenemase genes in a hybrid, conjugative plasmid of *K. pneumoniae* [159]. - Forty percent of assembled genomes from WWTPs discharged into rivers have both resistance and virulence genes [160]. - Antibiotics known to induce SOS responses that, in turn, promote HGT and can foster the mobilization of virulence genes in *S. aureus* [161].
Biofilm formation	- Antibiotics alter the expression of several virulence traits and biofilm formation [162].
Hypermutability	A trait often observed in pathogens from cystic fibrosis patients has been related to faster acquisition of AR, but hypermutators also have increased virulence, adaptation to airways, and transmissibility [163].
*GENE MOBILITY*	
Mobile elements	- The prevalence of integrons seems to be growing along with AR: from 0% in bacteria from the Murray Collection, to 3%/19% integron/resistance prevalence in the ECOR collection, to 26%/55% in the 2010s [164]. - Positive correlation between integron prevalence in *E. coli* and closeness to human activity [165]. - A 10-fold increase in AR genes borne by conjugative plasmids from 2000 to 2020 [166].
CRISPR-Cas	- Inverse association between multi-resistance, ESBL, and carbapenemase production and carrying CRISPR-Cas systems in *K. pneumoniae* [167]. - CRISPR-Cas systems are absent in most clinical isolates of the same species and, when present, lower resistance rates [168].

**Table 4:** Clinically relevant consequences of HGT, other than the mere movement of AR genes.

Consequence	Example
Accumulation of resistance towards “last resort” antibiotics within the same mobile element	- Colistin resistance *mcr-1* and carbapenem resistance *bla*_NDM-1_ [237].
“Accretion” of resistance and virulence genes	- Sequential acquisition of virulence ICE, multi-resistance plasmid carrying *bla*_NDM-1_, and chimeric plasmid carrying virulence and resistance determinants in *K. pneumoniae* [238].
Plasmid rearrangements	- Alternating fusion between three plasmids; the *mcr-8* gene can be co-transferred with a *bla*_NDM-1_ mediating carbapenem resistance or a *tmexCD1-toprJ1* gene mediating tigecycline efflux [239]. - ISs fuse AR gene-bearing, non-conjugative plasmids with non-AR, conjugative plasmids [240].
Gene amplification	- IS*26*-mediated amplification of beta-lactamase genes can foster resistance towards beta-lactams, including the newest combinations with beta-lactamase inhibitors [241].
Dispersal of “new” mobile resistance genes	- Tigecycline resistance genes *tet(X)* and *tmexCD-topJ* [242].
Simultaneous transfer of AR and virulence genes	- Carbapenem resistance and hypervirulence traits of *K. pneumoniae* by “vesiduction” [243] by fusion of plasmids [244,245] or co-conjugation [246].
Evolution of resistance plasmids	- *Acinetobacter* mega-plasmids, from carrying heavy metal resistance in those of environmental origin, to carrying AR genes in clinical isolates, by gaining transposons and integrons [247].
Reduction in fitness cost	- AR genes in ICEs that became inserted into glycan-synthesis genes in *Bacteroidales* are regulated by invertible promoters, diminishing the fitness cost of carrying such genes in the absence of antibiotics [248].
Reservoirs of AR genes	- IncX3 plasmids carrying the NDM-5 gene mediate conjugation from *E. coli* into Gram-positive *E. faecalis* and back to *E. coli*, making *E. faecalis* a reservoir of AR genes usually considered relevant when present in Gram-negatives [249]

**Table 5:** Unexpected places where AR genes and/or bacteria can be found.

Place	Description
Air	- Hospital air carries AR genes, with occupational exposure just as high as it is in farms, with 5.6 × 10^4^ gene copies inhaled during an 8 h shift [267]. - AR genes and bacteria can be found in urban air pollutants, especially in particulate matter 2.5 µm and smaller (PM_2.5_ [268]). - Airborne AR genes have been detected in pharmaceutical factories [269].
Dust	- The presence of AR bacteria in urban dust has been documented for quite some time (e.g., Ref. [270]). - Membrane vesicles in quantities ranging between 10^7^ and 10^11^ per gram of dust, and carrying AR genes, have been reported [271].
Garbage	- Landfills contain large amounts of AR bacteria and genes that can leak as leachates, becoming the greatest source of soil AR [272]. - Wasted food, ending up in landfills that generate leachates to soil and water bodies, also contributes to the dispersion of AR; decomposing vegetables modify the prevalence of AR genes in leachates [273].
Probiotics	- Korean probiotic formulations showed many contained multi-resistant strains in phenotypical assays, but only two AR genes were detected by PCR [274].
Planes and ships	- Bacteria in the sewage of airplanes have “an extraordinarily rich set of mobile AR genes” [275]. - Travel itself can be an important source of AR bacteria and/or genes: travelers suffer microbiome changes, enriching the content of AR genes [276]. - *Vibrio* spp. resistant to beta-lactams have been detected in ballast water [277].
Clouds	- AR genes have been detected in clouds at concentrations ranging between 1 × 10^3^ and 1.6 × 10^4^ copies per m^3^ of air [278]. This, along with bacterial deposition rates from the atmosphere to the soil of 3 × 10^6^ to >8 × 10^7^ per m^2^ per day [279], indicates a relevant route of dispersion of AR bacteria and genes.
Microplastics	- Microplastics act as reservoirs of AR bacteria in oceanic environments [280] and WWTP [281,282].
Zoos	Bacteria from zoo animals often carry resistance genes, making such animals and places, and the manure they generate, potential urban reservoirs of resistance [283,284].
Insects	- Flies carry AR organisms: - In cities, they carry ESBL-producing *E. coli* (12.9% in Berlin [285] and exactly the same in Mexico City [286]); in Laos, they even carry *mcr-1* and/or *mcr-3* [287]. - Flies can also transfer bacteria from farms to neighboring urban areas [288,289]. - Cockroaches carry *E. coli* harboring *bla*_CTX-M1_ and even *mcr-1* [290]. This could be particularly dangerous in hospitals [291].
Human cadavers	Saprobes, microbiota members, and, especially in victims of infections, pathogenic bacteria thrive in cadavers. Those microorganisms can be transferred to funeral workers and houses; then, they are released from body fluids into wastewater and, of course, from the whole cadaver or other solid wastes into the soil and water bodies. This “thanato-resistome” can then disperse into the environment and gain re-entry into the realm of the living through the usual ways (water, foodstuff, insects, rodents, etc. [292]).
Outer space	AR genes were detected on board the International Space Station [293].

**Table 6:** Non-canonical options for prevention and treatment of AR infections.

Option	Example
New uses of antibiotics	- Cycling within patients, guided by sequencing of low-frequency resistance mutations, can modify the evolution of resistance, especially in chronic infections [171]. - Use of “collateral sensitivity”, i.e., when gaining resistance to one drug increases susceptibility to another [364]; this concept was introduced in 1986 as “negative cross-resistance” [365].
Microbiome-modulating agents	Explored for the treatment of *C. difficile* infections [348].
Plasmid elimination	By direct chemical means, or by using “interference” plasmids with post-segregational killing systems [366,367,368].
Conjugation inhibition	- Anti-HIV drugs abacavir and azidothymidine [369]. - Inhibition of the establishment of AR genes after being acquired by conjugation [370].
CRISPR-Cas systems	- Used as plain antibacterials [371]. - Placed into conjugative plasmids [372] or transposons [373], and have been used to target virulence and AR genes. - For plasmid elimination [374].
Predatory bacteria	*Bdellovibrio bacteriovorus* has been proposed as a “living” antibiotic [375].
Inhibitors of bacterial membrane vesicles	Inhibitors of *Pseudomonas’* quinolone signal, peptidyl arginine deiminase inhibitors, or vesicle-stimulated inflammation inhibitors [376].
Traditional Chinese herbal medicine	Herbs or herb extracts with antibacterial activity against multi-resistant bacteria (mostly Gram-positives, but also *P. aeruginosa* and *A. baumannii*), alone or in combination with antibiotics [377].
Antibiotic adjuvants or potentiators	- Efflux pump inhibitors, modifying enzymes, membrane permeabilizers [378]. - Ascorbate diminishes the MIC of several antibiotics towards *E. coli* in synthetic human urine [379].
Lysostaphin	An enzyme that lyses the cell wall of *S. aureus* and/or nisin, a bacteriocin, to treat staphylococcal infections [380].

**Table 7:** Association between socioeconomic factors and AR.

Factor	References	Association
*INCOME MEASURES*		
Total GDP	[414]	None
GNI per capita	[410]	
High GDP per capita	[408]	
GDP per capita	[415,416] ^a^	Negative
GNI	[417,418]	
Income inequality	[419]	Positive
GDP per capita	[407,416] ^a^	
*GOVERNANCE*		
Corruption control	[407]	Negative
Lower corruption, political stability, rule of law, absence of violence	[408]	
Corruption control, rule of law	[414]	
Corruption control, voice and accountability, political stability and absence of violence/terrorism, government effectiveness, regulatory quality, rule of law	[420]	
Corruption perception (high perception = low corruption)	[410]	
Voice and accountability, government effectiveness, regulatory quality, rule of law, corruption control	[415]	
Higher levels of corruption	[409]	Positive
*HEALTH EXPENDITURE*		
Private health expenditure	[407]	Positive
Ration of private to public health spending	[408]	
Out-of-pocket expense, private health expenditure	[414]	
Out-of-pocket health expenditure	[416]	
Private expenditure on health (as % GDP)	[411]	
Total healthcare expenditure	[408]	Negative
GDP for health	[414]	
Health expenditure per capita	[411,415]	
*INFRASTRUCTURE*		
Improved infrastructure (sanitation, safe water, internet accessibility, urbanization, access to electricity)	[408]	Negative
Improving sanitation	[412]	
Unsafe water sanitation and hygiene	[410,420]	None
*ACCESS TO HEALTHCARE*		
Lower density of health facilities, larger numbers of beds in fewer facilities	[409]	Positive
Lower physician density	[416]	
Healthcare access or quality	[410]	Negative
Access to immunization obstetric care	[420]	

GDP: gross domestic product; GNI: gross national income. ^a^. Zhen et al. [416] found that GDP per capita was positively associated with AR in mainland China, especially with higher MRSA prevalence, but it was negatively associated in central and western zones in China, especially with *K. pneumoniae* resistant to third-generation cephalosporins.
